# Insights from the COVID-19 perspective on the necessity of corporate social responsibility in times of crisis in the context of Pakistan

**DOI:** 10.1371/journal.pone.0303675

**Published:** 2024-08-15

**Authors:** Sohail Ahmad, Qingyu Zhang, Zaheer Ahmad

**Affiliations:** 1 Research Institute of Business Analytics and Supply Chain Management, College of Management, Shenzhen University, Shenzhen, China; 2 Government College of Management Sciences Mardan, Mardan, Pakistan; University of Nottingham Ningbo China, CHINA

## Abstract

In light of the COVID-19 pandemic, there has been a notable increase in global awareness among businesses and consumers regarding environmental preservation and health concerns. The degree to which individuals identify with an organization is influenced by the appeal of its organizational identity, which aids in fulfilling important self-definitional needs. Nevertheless, there remains a limited understanding regarding the underlying factors that contribute to the phenomenon of firm identity attraction (IA) within the framework of consumer-business interactions. This study presents a validated framework that examines the antecedents of identity attraction, with COVID-19 being considered as a moderator. The framework is developed based on established theories of social identity and organizational identification. The study in Pakistan’s cities utilized a questionnaire survey as its primary research method, while the findings were evaluated through the application of structural equation modelling. The results of our study indicate that the impact of corporate social responsibility (CSR) on firm Identity Attraction (IA) is significantly more pronounced. Although the impact of COVID-19 on the intention to purchase remains unaffected, it does play a favorable role in moderating the influence of CSR on the attraction towards brand.

## 1. Introduction

While there has been an increase in customer-focused literature on firm interactions [1], the consumer’s perspective on these connections has remained untapped [2]. Productive customer relationship management necessitates an understanding of why customers join into business interactions in the first place. While it is obvious from the firm’s perspective that building a positive association with its customers will increase customer devotion and retention, resulting in increased brand profitability [3], customers’ inspiration to interact in an association with businesses is not always obvious. Customers’ desire to interact with businesses stems from their desire to assist them in meeting one or more essential self-definitional requirements through identity [4], and recent research has discovered that one factor in customers’ willingness to interact with businesses is to assist them in meeting one or more important self-definitional demands through identification [5, 6].

The concept of identification, which emerged from the field of organizational behavior and social psychology, addresses the human desire towards social identity and self-definition. Research has shown that identification can effectively enhance member engagement [7] as well as the citizenship standards of employees [8]. Social identity is influenced by organizations [9], the phenomenon under consideration can be described as the process of assimilating the values and behavioral standards of a collective entity, and incorporating a group identity into one’s self-perception.

Researchers and theorists claim that the attraction of the organizational identity determines the degree to which individuals identify with an organization [10]. Identity attraction (IA) is considered to be a critical component of identification in the consumer brand connection [6]. In spite of the significance of consumer-brand identification in the marketplace, little is known about the aspects that affect consumer IA.

Furthermore, scholars have posited that the theoretical lens of organizational identification theory could provide a robust framework for understanding the mechanisms through which CSR initiatives effectively foster proactive consumer assistance [11]. CSR connections show how an organization is doing and thinking in regard to its social responsibilities [12]. In most instances, it is the consumer who holds the power to determine the continuation or termination of a relationship with a business. However, the adoption of socially conscious business practices may foster a sense of customer identification [13]. Using this as a foundation, it’s important to consider how consumer ties to corporations relate to corporate reputation (CR) and corporate social responsibility [14], contribute to the generation of business IA.

On the assumption that customers will reward them for supporting social activities, several firms have embraced social causes [15]. In recent years, CSR has become a crucial academic concept and an important business agenda item [16, 17], even if it appears in big businesses as a number of concepts rather than a single notion [17].

Companies have been observed to partake in socially responsible endeavors not just to satisfy outward demands from stakeholders and comply with regulations, but also to gain from enlightened self-interest benefits including increased competitiveness and stock market performance [18]. Similar to how SARS in 2003 and the current COVID-19 pandemic have raised consumer and business awareness of CSR initiatives, especially those related to health and environmental protection. Therefore, it is crucial to comprehend the characteristics of consumer behaviors and CSR in order to study the effects of social responsibility in the challenging time of pandemic on customer response and purchase intention, as well as whether public health events play a moderating role in this impact.

Consumer sentiments about the firm are influenced by their understanding of CSR practices [19], corporate reputation [20], and brand appraisal [21], in promising ways. Beyond providing high-quality goods at competitive prices, consumers demand more from businesses [22]; they want corporations to exhibit social principles like community involvement.

However, traditional factors like price, quality, and brand familiarity seem to still be the most important selection factors in customers’ purchasing decisions [23], individuals still choose purchases for selfish rather than societal reasons [24]. Further study into how CSR affects consumer impressions is required in light of these seemingly at conflicting findings.

The objective of this study is to examine the impact of the COVID-19 pandemic on the association between CSR actions and consumer-based firm identity attraction (IA) within the domains of environmental preservation and health concerns. The objective of this study is to create and verify a theoretical framework that identifies the factors that lead to IA, with a specific focus on CSR, and examine how the COVID-19 pandemic influences this relationship. The study was carried out in Pakistan, utilizing a questionnaire survey and structural equation modeling. Its primary objective is to offer valuable insights into the changing dynamics of consumer-business interactions and their influence on IA. By doing so, it aims to contribute to a more comprehensive comprehension of the relationship between CSR, pandemic-related factors, and consumer perceptions within this particular context. Overall, our work contributes in a number of ways to the growing body of information concerning consumer-company identification relationships. First, our study further supports the notion that consumers not only utilize products, but also the businesses responsible for producing stated products, in order to fulfill their significant self-definitional desires, identity of the organization [25, 26]. Second, we provide evidence to demonstrate the association between IA and how customers perceive corporate relationships (both CSR and CR). We examine relationship marketing from the perspective of the customer. Finally, our model illustrates the potential impact of corporate social responsibility on identity attraction, a significant factor influencing consumer-brand interactions, through various mechanisms. The novelty of this article is to pay attention to Pakistani consumers and enterprises and the moderating influence of COVID-19 since the worldwide COVID-19 outbreak in 2020 has altered people’s purchase habits and perspectives on CSR.

The following sections comprise the division of the paper: The introduction is covered in the first section, while the literature evaluation and elaboration of hypotheses drawn from the most recent research are covered in Section 2. The design and research technique are presented in Section 3. The data analysis and hypothesis testing are presented in Section 4. The study’s conclusions, suggestions, and areas for future research are presented in Section 5.

## 2. Literature review and hypothesis

### 2.1. Identity attraction

The differentiation, services, and product offering that sets a firm apart from the competition and attracts potential customers is reflected in its brand identity attraction. What drives a firm is its brand identity attraction. It’s the primary contrast that makes a brand stand out from rivals in the industry [27].

According to scholarly investigations in the field of identity studies, individuals necessitate a robust and enduring sense of self-definition within a specific context [28]. Based on social identity theory, self-definitions are formed through the amalgamation of significant idiosyncratic traits, such as aggressiveness and ambition, alongside social identities, such as gender and occupation [25]. The establishment of self-definitions holds significance as it enables individuals to gain a comprehensive understanding of themselves and serves as a guide to avoid undesirable thoughts, actions, and emotions [29]. Individuals possess a significant drive to augment their self-esteem through the pursuit of positive social identities and the cultivation of favorable self-perceptions [30].

To provide insight on the process through which people identify with organizations, organizational identification and social identity theory have been combined [31]. Organization identity is a subjective viewpoint derived from the organization’s (perceived) essential, distinctive, and enduring qualities [32]. According to the research, perceived organizational identity is a substantial cognitive construct that influences the extent to which an individual within the organization identifies with it [33]. The degree to which an individual’s personal traits are congruent with the perceived organizational characteristics can be regarded as a manifestation of social identification [4]. The psychological relationship between individuals and organizations is of great importance in shaping various aspects, including the motivation of members to meet organizational requirements and objectives, their inclination to display managerial legitimacy and other collaborative conduct, and their likelihood of sustaining their affiliation with the organization [34].

Based on marketing research, it has been observed that consumers establish significance through their brand preference, selection, and consumption, thereby actively engaging in the construction or reinforcement of their personal identity [35], the degree to which a client sees a brand or organization as a partner [36], or as a reference group enhances the degree to which a customer recognizes with a business [37].

One of the elements that contribute to a customer’s interaction with a firm is the charm of its character. Similarity-Attraction Theory [38], Social Identity Theory [39], and Self Categorization Theory [40], all argue that individuals are inclined to, prefer, and promote associations with those that share similarities with them in order to augment their self-esteem and uphold a positive self-concept. Individuals who possess comparable beliefs, attitudes, hobbies, or experiences tend to engage in more effortless and seamless interactions, characterized by a reduced cognitive effort [41].

Identity attraction is the degree to which individuals exhibit positive attitudes, are drawn to, and actively endorse affiliations with a company, primarily influenced by its enduring characteristics [42]. The capacity of a business to attract consumers is contingent upon its ability to fulfill fundamental criteria that consumers utilize to define themselves [6], self-continuity refers to the inclination of individuals to differentiate themselves from others in various social settings by aligning themselves with a business that possesses a unique principles, approach or other characteristic attributes. Self-distinctness refers to the desire to seek out a business entity that shares a comparable identity to one’s own. This inclination arises from the desire to associate with a corporation that possesses a perceived identity that is appealing, with the aim of enhancing one’s self-esteem by receiving a more favorable evaluation of oneself.

### 2.2. Consumer brand congruity

Consumer brand congruity is a concept that pertains to the extent to which a consumer’s self-concept aligns with the perceived identity of a brand [43]. Consumers and brands have a complex relationship that involves elements like trust, nostalgia and previous experiences, perceived quality of interactions, and a consumer’s sense of self-connection with a brand [43]. More specifically, consumers choose brands based on their self-concept-enhancing symbolic meanings as well as functional/utilitarian attributes. Consumer’s self-connection with a brand also known as consumer-brand congruity influences brand choice [44]. The significance of the alignment between consumer brand congruity and a company’s identity attraction is noteworthy. Consumers are more inclined to be attracted to a brand when they perceive a congruity among the brand’s values, personality, and image and their own self-concept [45]. The alignment between the consumer’s identity and the brand’s identity fosters a feeling of resonance and relatability, resulting in heightened emotional attachments and increased brand loyalty. Therefore, the integration of consumer brand congruity into a company’s marketing strategies can result in the enhancement of its identity attraction, the cultivation of long-term customer relationships, and ultimately the attainment of a competitive advantage in the marketplace [46].

According to cadiz et al., [47], the "Attraction-Selection-Attrition model", individuals are inclined to be attracted to organizations that exhibit similarities between their personal characteristics and the attributes of the organization. Individuals seeking employment often choose employers with whom they perceive a compatibility between their own core values and those upheld by the organization [48]. A person is more likely to be lured to firms that they think would fit them during the hiring process [49].

Both traditional identity consumption theory and its counterpart emphasize the significance of identity similarity and attraction in shaping consumer perceptions, choices, and decision-making [50], and the more contemporary approach for consumer-company identification [6]. The similarity-attraction paradigm, which holds that people gravitate toward others with whom they share characteristics, serves as the foundation for these two contributions [38]. Individuals tend to gravitate towards, exhibit a preference for, and provide support to individuals who share similar characteristics in order to enhance their self-esteem and preserve a sense of balance in their self-identity [51, 52].

Consumer brand congruity is the degree of alignment between a company’s brand image and the expectations held by its target consumers [46]. This alignment has been found to have a significant and positive influence on the extent to which consumers are attracted to a company’s identity. When a business consistently fulfills its commitments and aligns with the values and preferences of its intended audience, it cultivates a perception of trustworthiness, dependability, and alignment [53]. The alignment between consumers and the company is reinforced by this congruity, as it leads consumers to view the brand not solely as a source of products or services, but rather as a manifestation of their own values and goals. The establishment of a stronger connection and alignment between consumers and a company results in heightened consumer loyalty, positive word-of-mouth, and a more profound emotional attachment to the firm [6]. Consequently, this enhances the company’s attractiveness in terms of identity for consumers, potentially leading to higher brand advocacy and long-term business success. As a result, we provide the following hypothesis:

**H1**: The higher the sense of consumer-brand congruity, the more appealing the company’s identity is to consumers.

The correlation between identity attraction and purchase intention pertains to the impact of a consumer’s affiliation with a particular brand or product on their propensity to engage in a transaction [54]. Identity attraction refers to the phenomenon wherein individuals perceive a resonance between their own values, personality traits, or overall identity and those projected by a brand [55]. Purchase intention, conversely, refers to the quantifiable assessment of an individual’s inclination to acquire a specific product or service. The presence of a strong sense of identity attraction towards a brand has the potential to exert a positive influence on consumers’ purchase intention [56]. This implies that when individuals perceive a brand as congruent with their self-concept or desired identity, they are more inclined to indicate an intention to engage in a purchase from that particular business [57]. The capacity of a business to foster a robust sense of identity appeal among consumers can significantly enhance their intention to make a purchase [58]. When individuals have a profound and significant affiliation with the corporate identity of a firm, there is an increased likelihood of cultivating trust, loyalty, and a shared value system with the brand. The emotional resonance and alignment with the company’s values not only serve to enhance the overall perception of the business, but also exert a substantial influence on the consumer’s desire to make a purchase of its products or services [59]. The presence of a robust identity attraction establishes a connection that surpasses the mere offering of a product or service, hence cultivating a preference for a particular business and frequently resulting in heightened consumer loyalty and endorsement. Consequently, enterprises that effectively cultivate identity attraction within their target audience can ultimately enhance their financial performance by stimulating more intention to purchase, thereby leading to increased sales and a larger market presence. There are multiple factors that contribute to this relationship. To begin with, the act of identifying with a business serves to strengthen the bond between consumers and the business, thereby cultivating a feeling of trust and fostering loyalty [60]. Additionally, when individuals establish a sense of identification with a business, they perceive the products or services offered by the brand as aligning with their desired self-image, thereby resulting in an increased intention to make a purchase [61]. Finally, the phenomenon of identity attraction serves to enhance individuals’ feelings of affiliation and attachment to a brand’s community, thereby potentially bolstering their inclination to make purchases [62]. Brands frequently exploit this association by employing branding strategies that highlight mutual values, customized encounters, and focused communication. Through the strategic utilization of consumers’ identity and the cultivation of a sense of belonging, brands possess the ability to exert a favorable impact on purchase intentions. In this sense, one may hypothesis a link between identity attraction, and purchase intention.

**H2:** Identity attraction has a direct positive impact on purchase intention.

### 2.3. CSR associations

Over the past decade, numerous scholarly investigations have been conducted to examine the degree to which consumers are impacted by their engagements with a company’s corporate reputation (CR) and corporate social responsibility (CSR) [63, 64]. CSR affiliations function as a manifestation of an organization’s perspective and its engagement in activities related to societal responsibility [65]. Because of its unique and special underpinnings, the business’s character as expressed through its CSR deeds is not only fundamental and frequently long-lasting, but also typically more distinctive than CR elements of the corporate structure [66].

Customers have a higher emotional reaction to certain businesses and brands than others [67]. Consumers are more likely to hold the belief that a company possesses specific desirable attributes that align with their own self-perception when said company conducts its operations in a manner that is perceived as socially responsible [68]. According to gioia et al., [69], certain businesses employ a strategy wherein they actively involve their clients in a specific social movement, thereby positioning themselves as prime examples of broader categories. These organizations are endeavoring to enhance their credibility by embracing attributes that they perceive as highly valued by stakeholders. Consumer brand congruity (CBC) refers to the perceived similarity between a company’s character and that of consumers. The strength of CBC is positively influenced by a company’s engagement in CSR activities. This is because such actions signal to consumers that the company possesses attributes that align with their own values, such as being civic-minded, compassionate, and socially active. Put simply, it is more straightforward to identify a notable alignment in values among companies that are regarded as socially responsible [70]. As a result, we propose:

**H3**: Consumer-brand congruity is more positively correlated with consumer perception of CSR.

The influence of CSR on the attraction of identity for individuals and communities is substantial [12]. When a corporation actively participates in CSR initiatives, it showcases a dedication to upholding ethical standards, promoting sustainability, and fostering social welfare [71]. This is consistent with the values and beliefs held by a significant number of consumers and employees, resulting in a favorable perception of the company’s corporate identity. Individuals are inherently inclined towards affiliating with enterprises that prioritize CSR due to its manifestation of a sincere commitment towards environmental preservation, societal well-being, and the interests of stakeholders that extend beyond mere profit generation [72]. These companies not only draw in consumers who are inclined to support ethical brands, but also appeal to individuals seeking purposeful employment and desiring to work for organizations that make a positive impact. In essence, CSR engenders a profound sense of affinity by cultivating trust, allegiance, and a collective objective among diverse stakeholders.

From a marketing viewpoint, the relationship between positive product and brand assessments, brand preference, and brand referrals among consumers may be used to identify the company’s financial benefits from CSR [65, 66]. According to the research, CSR initiatives "may impact customers’ overall wellbeing without necessarily translating to company-specific advantages." That contentment may be related to the corporate IA, since consumers’ self-esteem might increase when they identify with a business that engages in CSR activities [66] as a consequence of collaboration (association) with a socially responsible business [66]. In an alternative formulation, it is worth noting that despite the absence of perceived alignment between the consumer’s personal attributes and those associated with the brand, there exists a potential inclination to establish connections with companies engaged in CSR initiatives. This inclination, indicative of a heightened level of identity attraction (IA), may stem from the consumer’s recognition of the potential benefits derived from associating with the company, particularly in terms of fostering a sense of distinctiveness and personal growth. Customers are more likely to believe a company has certain qualities that align with their sense of self when it operates in a responsible manner in society [13]. As a result, we propose:

**H4**: The greater the customer’s sense of CSR connections, the more attractive the brand’s identity is to the customer.

### 2.4. Brand assessment

Brand assessment is the measure of how positively or negatively the subject views the company as a whole. This overall evaluation is based on the company’s core, defining, and long-lasting characteristics, which are crucial components in shaping the identity and reputation of the firm [73]. It shows that the organization has the respect and adoration of relevant referents[74, 75]. The individual fulfils their self-esteem and security needs, which are among the three fundamental self-definitional prerequisites by increasing his or her social standing [76], access to definite social prospects [77], or just the belief that by retaining connections with a business, he or she is "basking in reflected glory" [78]. Therefore, the more well-known a firm is, the greater the likelihood that a consumer would feel more positive about themselves by relating to it [76]. Given that evaluation leads to prestige [73], and that customer association with the brand has been connected to identity prestige [42], high levels of the company’s IA will be the consequence of a positive evaluation. As a result, we’ve come up with this hypothesis:

**H5**: The more favorable the consumer’s assessment of the brand, the better the company’s identity attraction.

Organizational preferences, work happiness, organizational commitment, and turnover intentions are all positively impacted by person-organization congruence, according to studies on organizational identity [79]. Research on the evaluation of brand extensions in the context of consumption has often shown that there is a greater impact of parent brand associations on consumers’ assessments of new products when they believe the product and the brand are well matched [80]. How comparable they are having to do with how well the old brand and the new goods or services are considered to fit together. On the other side, customers who notice a discrepancy have a poor opinion of the business and its operations [81].

Customers will regard a company more favorably as a result of CBC since they will be more devoted to it. According to bhattacharya and sen [6], The effect of CSR on a consumer’s BA is probably mediated through CBC. Sen and bhattacharya [66], used regression analysis to assess the proposed connection, and it was limited to strong CSR support consumers. We contend that CBC results from the overlap of customer and business character traits other than those that are directly acquired through CSR links, indicating that there is a more widespread (and not limited to customers who strongly favor CSR) influence (examples of qualities that can contribute to the achievement of goals or maintaining a presence in a market include persistence.). Furthermore, it can be argued that in conjunction with corporate social responsibility in customer service, an elevated consumer brand congruity (CBC) will consequently contribute to an increased brand assessment (BA). This is due to the fact that a stronger alignment between individuals and the organization leads to a more positive perception of the company [82]. Because the customer and the firm’s character attributes intersect, it will implicitly reflect a more positive evaluation of the consumer himself. The following is what we propose:

**H6**: The higher levels of consumer-brand congruity are associated with higher levels of brand assessment.

According to the existing body of literature, consumers form their perceptions of organizations by considering two key factors: performance-based business associations and perceived social responsibility [83]. More specifically, a company’s ability-based reputation has a significant impact on total corporate rating [65]. The corporate reputation and brand assessment are intricately interconnected and exert a reciprocal influence on each other. The evaluation of a business’s reputation, which encompasses factors such as its ethical conduct, transparency, and social responsibility, has a substantial influence on how consumers and stakeholders perceive the brand [84]. A favorable corporate reputation contributes to the assessment of a brand by cultivating trust, loyalty, and credibility within the market [85]. The effective management and protection of corporate reputation are of paramount importance in order to cultivate a favorable brand assessment and maintain long-term prosperity within the fiercely competitive business environment. Hence, we developed the following hypothesis.

**H7**: The greater the CR, the more favorable the consumer’s assessment of the brand.

It is crucial to stress that in the business areas, which are characterized by a greater degree of perceived risk, a positive reputation motivates the consumers to pick the firm or brand, at the expense of alternative products that would otherwise be equivalent in regards of price or quality [86]. Burlea-Schiopoiu and Balan [87], came to the opinion that reputation has an effect on stakeholders’ involvement. As a result, corporate reputation and the dissemination of information about the company were deemed by Caruana et al., [88], to impact the customer’s purchasing behavior through their effects on perceptions concerning the value of the items. According to this concept, a company’s reputation reduces the risk that consumers perceive and motivates them to establish commercial connections with it [89, 90].

The concepts of corporate reputation and consumer brand congruity are intricately linked and exert a substantial influence on the achievement of a business. Corporate reputation encompasses the collective perception and evaluation held by consumers regarding a company, which is derived from previous conduct, behaviors, and overall operational effectiveness [91]. In contrast, consumer brand congruity pertains to the uniformity and coherence between the brand image of a corporation and the anticipated beliefs and principles of its intended consumer base [43]. The existence of a robust correlation between corporate reputation and consumer brand congruity can result in various favorable consequences. A favorable corporate reputation has the potential to increase consumer trust and credibility, thereby reinforcing the alignment between the brand and the consumer [92]. This alignment cultivates brand loyalty, favorable consumer attitudes, and ultimately, heightened customer preference and intention to make a purchase. On the other hand, an unfavorable corporate reputation or a lack of alignment between a brand and consumer expectations can lead to the gradual decline of trust, impairment of brand perception, and the possible attrition of customers. Hence, it is imperative for businesses aiming to achieve long-term success in the market to prioritize the establishment and preservation of a favorable corporate reputation, alongside the maintenance of brand congruity. In line with the previous findings, the following hypothesis is formulated:

**H8**: The greater the CR, the more favorable the consumer-brand congruity.

### 2.5. CSR support

The consumer’s social side urges him or her to avoid purchasing things from firms that are harmful to society and to proactively pursue the acquisition of products from businesses that are beneficial to society [93]. "The socially conscious consumer examines the public consequences of his or her private consumption or aspires to use his or her purchasing power to effect social change," [94]. Customers weigh their thoughts on firm’s ethical or immoral activities while making purchases [95]. Customers want businesses to act ethically, and they are willing to hold them accountable if they don’t [96].

Consumers who have high levels of support to a company’s CSR domain will see more similarities or a shared resemblance between themselves and the company than customers who have low levels of trust in that domain [66]. Customers who trust a company’s CSR efforts highly value those programs. In order to find characteristics that are comparable to their personality traits in firms that engage in CSR activities, they will examine the company’s character traits that aren’t directly related to CR. If support is less, the customer will be less conscious of the qualities they have in common with the company. As a result, we recommend that:

**H9**: The greater the consumer’s CSR support, the stronger the consumer’s perception of consumer–brand congruity.

### 2.6. The moderating role of COVID‑19

The new coronavirus, known as COVID-19, first appeared in late 2019 and quickly spread over the world, sparking a pandemic. Its effects on public health, economy, and daily life have been extensive on a global scale. It quickly spread over the world, growing uncontrollably and having a negative impact on all economies [97]. Because of the government’s response to the epidemic, which includes neighborhood lockdowns, mobility limitations, stay-at-home orders, and social distancing laws, it is not only a health issue but also affects corporations and the world economy in various ways [98], and it has also sparked debate among certain academics on how it relates to CSR [99]. The study conducted by Ding et al., [100] examined the impact of CSR on the performance of companies amidst the COVID-19 pandemic. The findings of their research revealed that CSR initiatives play a significant role in attracting the attention and support of stakeholders, including consumers. According to the research conducted by Manuel and Herron [101], businesses are engaged in various forms of charitable CSR initiatives amidst the pandemic, potentially driven by utilitarian and moral rationales to meet the demands of both internal and external stakeholders. This phenomenon potentially resulted in an enhancement of corporate stock returns or a deceleration of the decline in corporate stock values. Bae et al., [102] hold the opinion that businesses that are open to social responsibility are not immune to the negative consequences of the crisis brought on by the COVID-19 outbreak if the CSR rating is out of line with real activity. Huang and Liu [103] posit that an examination of hotel CSR marketing reveals that the implementation of donation appeals during the COVID-19 pandemic yields a positive impact on the brand loyalty of businesses. According to Zhang [104], consumers’ perceptions of dairy CSR throughout the COVID-19 era would result in internal reactions including identity emotion, consumer trust, business reputation, and perceived product quality, as well as exterior reactions like purchase intention. According to this research, public health initiatives like COVID-19 will increase consumers’ awareness of CSR and its impact. During the COVID-19 pandemic, there has been a heightened global awareness of environmental preservation and health concerns [105]. As a result, consumers are more attuned to a company’s CSR efforts, particularly when they align with the current societal values and concerns magnified by the pandemic.

Social identity theory posits that individuals categorize themselves into social groups, including organizations, to enhance their self-concept and self-esteem [106]. When a business’s CSR activities resonate with these heightened societal values and concerns, it can serve as a powerful tool for enhancing the company’s organizational identity and strengthening consumers’ identification with the business [107]. During the pandemic, consumers may be more inclined to recognize and appreciate a company’s efforts in CSR, seeing it as a positive and socially responsible response to the current challenges.

This heightened alignment between a company’s CSR initiatives and the prevailing concerns intensified by the pandemic could create a more favorable context for identity attraction and, consequently, influence purchase intention. The hypothesis, therefore, suggests that COVID-19 positively moderates the impact of CSR, as it is expected to enhance the relationship between CSR, identity attraction, and purchase intention during this unique and contextually relevant period. In summary, we proposed.

**H10:** COVID-19 positively moderates the impact of CSR on identity attraction.
**H11:** COVID-19 positively moderates the impact of identity attraction on purchase intention.

**Fig 1:**
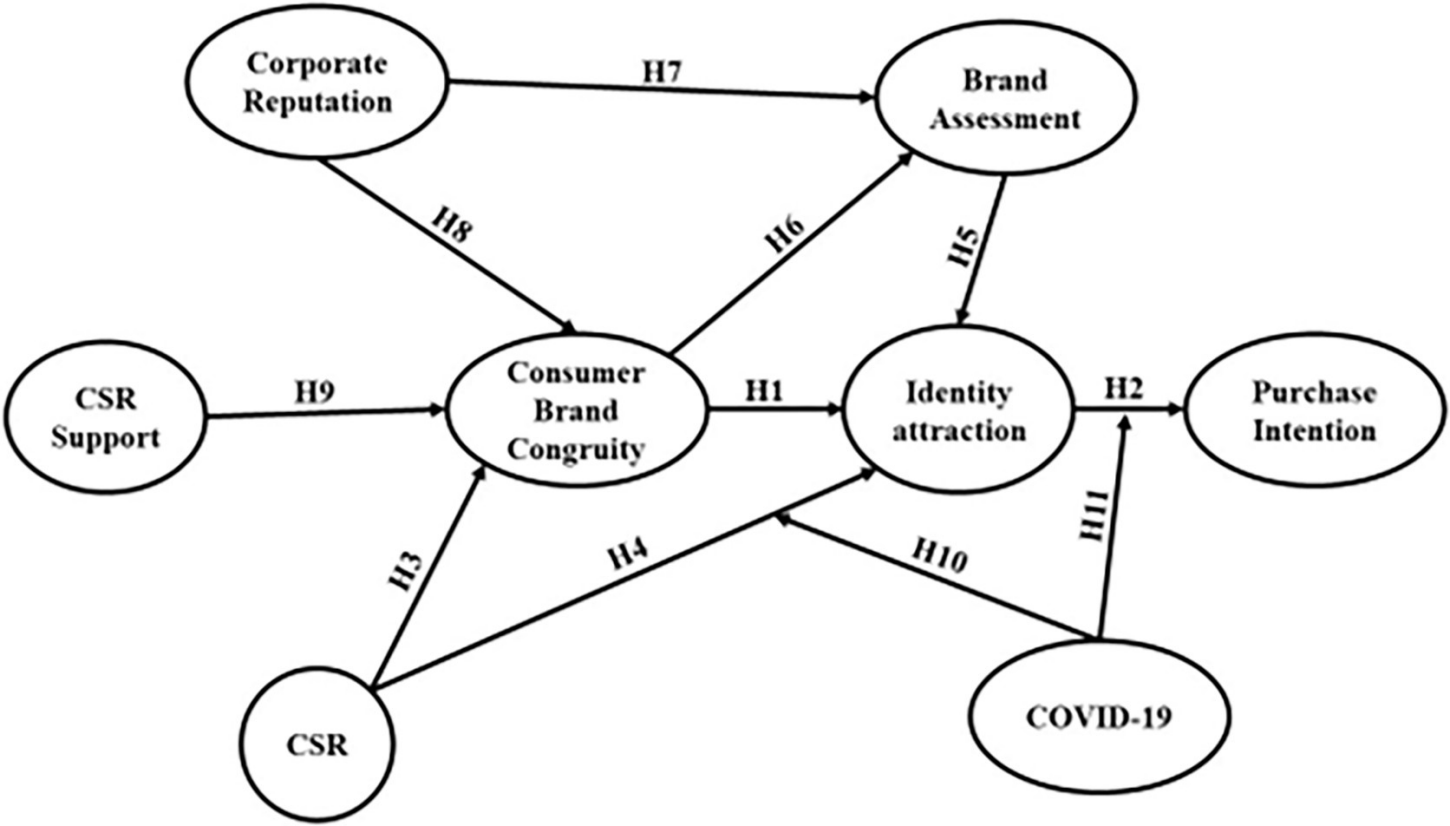
Conceptual framework.

The diagram depicting the potential relationships is presented in Fig 1. It is widely believed that the corporate affiliations of a company and the level of trust that its customers place in its corporate social responsibility initiatives are the primary factors influencing its identity attraction. Furthermore, apart from being a direct outcome of CSR connections, these benefits are also influenced by brand assessment and consumer-brand congruity, which can be attributed to the favorable social perception associated with these associations. Customers’ opinions of a company’s value and how closely it resembles them influence whether they prefer, are drawn to, and support relationships with it.

## 3. Methodology

### 3.1. Instruments development

For assessing the constructs, we employed multi-item measures that have previously been used in research. All components were assessed using a five-point Likert scale. The 5 CSR items were assessed using a framework established by [54, 108], 3 elements were amended to assess brand-consumer congruity from [43, 109], 4 corporate reputation items have been adopted from [110], 4 items were adjusted from [82, 111], for CSR support; 6 items were modified from [82], for brand assessment; 4 items from [54, 112], for Identity attraction. 3 items were modified from [108], and items for covid-19 were taken from [103, 104]. Table 1 shows the items.

**Table 1:** Construct and their respective items.

Construct	Items	Sources
**Consumer-brand congruity**	The way I view myself is congruent with how I use X in my daily life. My regular use of X reveals who I am. X is used by people like me every day.	[43, 109]
**Corporate social responsibility (CSR)**	X is highly concern for local communities. X is highly concern for environment. X is very concerned about corporate donations to deserving charities. Women’s concerns are very important to X. Concerns for the disabled minority are of great importance to X.	[108]
**Corporate Reputation (CR)**	In the sector, X is a pioneer. X is a business that excels at technical innovation. X provides a top-notch product. X provides excellent customer service.	[110]
**CSR supports**	As a client of X, I concur that a portion of its efforts are directed toward promoting the inclusion of underrepresented and underprivileged groups. X Take steps to safeguard the environment. X Contribute to social justice initiatives. X Support groups that stand up for sports and culture.	[82, 111]
**Brand assessment (BA)**	X is a company with a solid reputation. X is a financially sound organization. X is a company I believe in. I believe that X is a long-term-focused business. I believe that in five years, X will still be in operation.	[82]
**Identity attraction (IA)**	X is an extremely appealing company. I prefer X because it stands apart from the other financial institutions. Because I can tell that X understands me, dealing with X makes me feel wonderful. Its reputation as a premium brand is well known.	[54, 112]
**Purchase Intention**	I’ll keep thinking of the business as my primary brand. I would continue to use this business’ services. If someone sought my opinion, I would advise them to use the company.	[108]
**COVID-19**	The business should follow the society’s pandemic prevention strategy during COVID-19. The company should take on the appropriate charity duty during COVID-19. The business should be held accountable for its ethical behavior toward its stakeholders during COVID-19. The company should take a proactive role in the community’s environmental preservation initiatives during COVID-19. I would consider buying from the firm if they show greater social responsibility during the COVID-19 pandemic.	[103, 104]

### 3.2. Sample and data collection

We selected to gather data in Pakistan using a paper questionnaire survey targeting Pakistani CSR-engaged customers since Pakistan is a developing country with a growing awareness of CSR. Our sample frame consists of CSR-engaged consumers from 4 major cities of Pakistan including Islamabad, Peshawar, Mardan, and Lahore. The data was collected in accordance with the Declaration of Helsinki, and approved by the Ethics Committee of Shenzhen University (January 2022). During the administration of questionnaires to the designated sample, the researcher provided a comprehensive explanation of the study’s aims and obtained informed consent (verbal) from participants prior to their engagement in the survey. The survey is optional, and participants have the freedom to withdraw their participation at any point during the process. The author clearly stated that the data would be used solely for research purposes and would be kept confidential. A convenience sampling approach was utilized in several cities to assure greater representation. Despite the fact that many academics doubt the generalizability of convenience sampling findings, there is evidence to show that using educated and urban residents for this type of study is accurate [113]. The study’s target group was educated urban customers since they were more likely to reply to the survey due to their greater understanding of CSR [114, 115]. Islamabad, Peshawar, Mardan, and Lahore were the four cities from which we gathered data. There were two sections to the questionnaire. Gender, age, education, and income were all factors evaluated in the first half. Individuals from various colleges, universities, government offices, superstores, media outlets, hospitals, restaurants, hotels, and corporations are randomly visited by data collectors. The information was gathered during February to April of 2022. A total of 700 questionnaires were sent out, and 647 people responded. Because 65 replies were missing, data cleaning resulted in 582 viable surveys. There were 310 male respondents and 572 female respondents, with the majority of the respondents being between the ages of 21 to 40 and can be seen in Table 2. The most frequent participants were those with bachelors and master degrees. The poll included 33 questions that were ranked on a five-point Likert scale extending from strongly disagree (= 1) to strongly agree (= 5). The measurements and structural models were tested using SmartPLS.

**Table 2:** Number of respondents.

Number of respondents		Frequency	*Percentage*
Gender	Male	310	53.26%
	Female	272	46.73%
Age	18 to 30 years old	211	36.25%
	31 to 40 years’ old	165	28.35%
	41 to 50 years old	130	22.33%
	51 years old and above	76	13.05%
Education	Primary Education	55	9.45%
	Middle School	83	14.26%
	High School	96	16.49%
	Bachelor Degree	113	19.41%
	Diploma/Certificate	88	15.12%
	Postgraduate Degree	147	25.55%
Profession	Students	245	42.09%
	Self Employed	117	20.10%
	Job Holders	118	20.27%
	Businessmen	102	17.52%
Income	0–15000RS	85	14.60%
	15,0001 RS-30,000RS	197	33.84%
	30,001 RS–50,000 RS	190	32.64%
	50,001 RS-80,000 RS	58	9.96%
	80,001 RS-100,000RS	32	5.49%
	More than 100,000 RS	20	3.43%

## 4. Data analysis and results

### 4.1. Techniques

The utilization of the covariance-based structural equation modeling (CB-SEM) approach has been observed in past decades for the purpose of conducting intricate analyses involving latent and observable variables. Moreover, it was observed that by the year 2010, a greater number of academic papers were employing the use of covariance-based structural equation modeling (CB-SEM) as opposed to partial least squares structural equation modeling (PLS-SEM) in their research endeavors. In recent years, there has been a notable increase in the quantity of papers utilizing PLS-SEM, surpassing the quantity of articles employing CB-SEM [116].

The Partial Least Squares Structural Equation Modeling (PLS-SEM) approach is commonly employed in academic research publications to estimate intricate models comprising numerous constructs, structural pathways, and indicator variables, while avoiding the imposition of assumptions on the distribution of data. Moreover, Partial Least Squares Structural Equation Modeling (PLS-SEM) is a variant of Structural Equation Modeling (SEM) that relies on the estimation of causal-predictive relationships using statistical models [117, 118]. Several authors provide comprehensive explanations and rationales regarding the utilization of the Partial Least Squares Structural Equation Modeling (PLS-SEM) approach, elucidating its appropriate application and timing [119, 120].

Numerous research studies have utilized the Partial Least Squares Structural Equation Modeling (PLS-SEM) approach to estimate complex models consisting of multiple concepts, structural paths, and predictor variables, while avoiding assumptions about the distribution of the data. Partial Least Squares Structural Equation Modeling (PLS-SEM) is a statistical approach that combines causal and predictive elements within the framework of Structural Equation Modeling (SEM). PLS-SEM places particular emphasis on prediction while estimating an empirical model, incorporating features that are specifically designed to provide potential explanations for observed phenomena [121, 122]. Thus, the method eliminates the potential for a conflict between prediction and explanation, which is necessary for developing management implications [123]. As a result, user-friendly statistical software, such as SmartPLS and PLS-Graph, is accessible that needs little technical understanding of the procedure [124]. PLS-SEM is deemed the most suitable method to employ in cases where the path model incorporates one or more formatively measured structures, the sample group is constrained due to a small population, the structural model is comprehensive and encompasses numerous concepts, the research primarily relies on secondary sources, and when the data distribution exhibits normality concerns.

The principle benefit of utilizing PLS-SEM is the unwinding of the typical dissemination suspicions needed for the greatest probability strategy utilized in assessing models utilizing CB-SEM, and the capacity of PLS-SEM to handily appraise more confounded models dependent on a small sample group [125]. PLS-SEM gives solutions for limited sample sizes when models include a large number of elements and constructs. Papers published recently [126], describe how PLS-SEM may solve problems when other approaches, such as CB-SEM, fail to converge with limited sample numbers and complicated models. Furthermore, several recent research shows that the lack of distributional assumptions is the primary rationale for using the PLS-SEM approach [127, 128].

Given the foregoing reasoning, we can conclude that PLS-SEM is well suited to this proposed study, particularly because when a goal is exploratory research for theoretical approaches; when the assessment is predictive; when the conceptual model is complicated; when the allocation lacks normality; and when the study necessitates a latent variable score for further analysis. The SmartPLS (version 3.2.1) was used to process the data.

PLS was used to analyze the data since that was better than the other structural equation models [129]. It also has the ability to compute dynamic models with various relationships [130], since the objective of population estimates is unknown [131]. PLS offers two integrated versions: an internal and an exterior version. The internal model analyses relationships among latent variables, whereas the external model, emphasizes similarities among latent variables and their indicators [132]. PLS, on the other hand, offers the slimmest loads when it comes to variable positioning. As a result, the bias caused by software covariance techniques is reduced by PLS [133]. To monitor the statistical relevance of path coefficients, we employed the bootstrapping procedure. In the verified model, all concepts were identified as meditative since their measuring aspects demonstrated these ideas.

### 4.2. Measurement model

While most measuring items are based on past research, an assessment of the measuring items’ validity and reliability is required to confirm the survey instruments’ precision and quality. The measurement model was examined using both convergent and discriminant validity. Convergent validity was assessed using four different methods of chin [133], (1) indicators having factor loadings 0.7 or higher are considered highly satisfactory [134]. While loading value of 0.5 is regarded as acceptable, the manifest variables with loading value of less than 0.5 should be dropped [133, 135].; (2) composite reliability (CR) having scores above 0.7; (3) Cronbach’s alpha scores; and (4) average variance extracted (AVE) estimates having scores above 0.5. Which are shown in Table 3. The model showed enough convergent validity based on the three measures. The findings of the measurement model showed that the model had good reliability, convergence validity, and discriminant validity and confirmed that the constructs were statistically diverse.

**Table 3:** Factor loadings, CA, rho_A, CR, AVEs.

	Items	Loadings	rho_A	CR	(AVE)	CA
Consumer-brand congruity	CBC1 CBC2 CBC3	0.795 0.815 0.804	0.846	0.846	0.647	0.846
(CSR)	CSR1 CSR2 CSR3 CSR4 CSR5	0.687 0.691 0.782 0.723 0.747	0.867	0.865	0.518	0.865
Corporate Reputation (CR)	CR1 CR2 CR3 CR4	0.813 0.890 0.806 0.842	0.879	0.904	0.703	0.860
CSR support	CS1 CS2 CS3 CS4	0.748 0.814 0.831 0.788	0.813	0.873	0.633	0.807
Brand assessment (BA)	BA1 BA2 BA3 BA4 BA5	0.798 0.814 0.757 0.776 0.609	0.814	0.867	0.569	0.807
Identity attractiveness (IA)	IA1 IA2 IA3 IA4	0.746 0.813 0.830 0.787	0.812	0.872	0.632	0.806
Purchase Intention	PI1 PI2 PI3	0.756 0.781 0.857	0.874	0.903	0.609	0.871
COVID-19	C1 C2 C3 C4 C5	0.888 0.865 0.797 0.771 0.804	0.860	0.899	0.692	0.851

### 4.3. Discriminant validity

Discriminant validity (DV) relates to the extent whereby the measures employed in a model exhibit distinctiveness from other constructs. In previous studies, researchers employed various techniques, including cross-loadings and the Fornell-larcker criteria to asses DV. Nevertheless, Henseler et al. [126], identified a deficiency in the DV and observed a relatively lack of sensitivity from these criteria. Hence, they provided a novel criterion called heterotrait-monotrait ratio (HTMT) to evaluate the DV of constructs, offering a more refined and sensitive approach. HTMT criteria states that constructs can be considered non-interchangeable if the correlations between their items are below 0.90. In order to assess discriminant validity, Henseler et al. [126], criteria of HTMT ratio were established for proposed research model; Table 4 shows the correlation value for all of the underlying constructs are below 0.90.

**Table 4:** Heterotrait-monotrait ratio (HTMT).

	CBC	CSR	CR	CSR support	BA	IA	PI	COVID-19
**CBC**	**….**							
**CSR**	**0.444**							
**CR**	0.302	**0.838**						
**CSR support**	0.343	0.162	**0.807**					
**BA**	0.202	0.634	0.733	**0.754**				
**IA**	0.021	0.031	0.010	0.041	**0.183**			
**PI**	0.378	0.183	0.528	0.348	0.218	**0.781**		
**COVID-19**	0.274	0.354	0.312	0.815	0.326	0.365	**0.832**	**….**

### 4.4. The structural model

**Fig 2:**
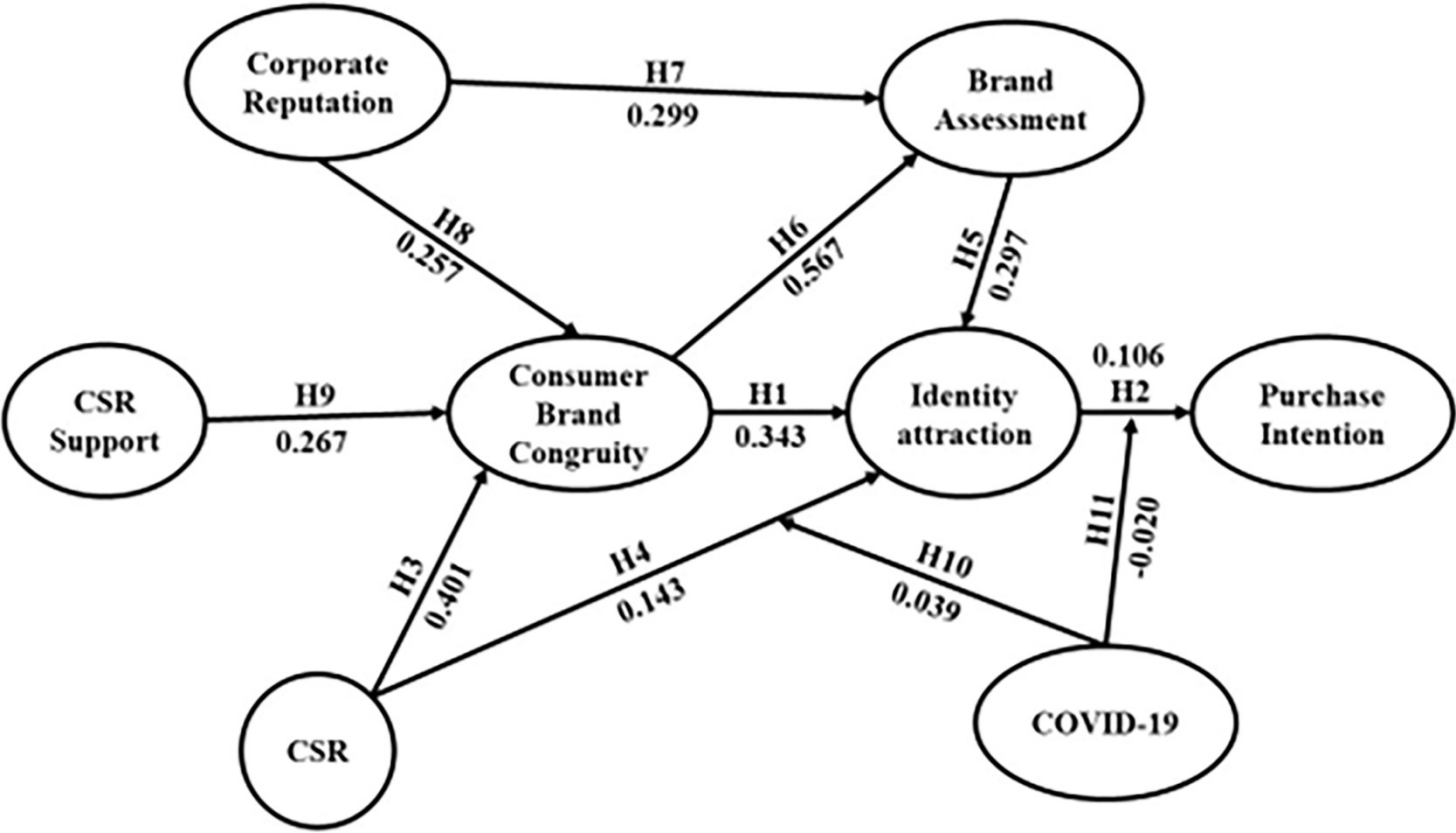
Structure model results.

The path coefficients pertaining to the structural model and the corresponding variants are depicted in Fig 2.

### 4.5. Path coefficient and confidence intervals

In addition, the analysis supported all of the major hypotheses. Similar to preacher and hayes [136], For the indirect effects, a 95 percent confidence interval was determined using a 1,000 re-samples bootstrap. Table 5 describes the PLS bootstrapping results.

**Table 5:** Results of path coefficients and confidence interval.

	Original Sample (O)	Sample Mean (M)	Standard Deviation (STDEV)	T Statistics (|O/STDEV|)	P-Values	Confidence Intervals		Result
						2.5%	97.5%	
CBC→ IA	0.343	0.346	0.025	13.527***	0.000	0.288	0.384	Supported
IA → PI	0.106	0.110	0.029	3.611***	0.000	0.046	0.161	Supported
CSR →CBC	0.401	0.403	0.047	8.457***	0.000	0.310	0.492	Supported
CSR →IA	0.143	0.146	0.029	4.930***	0.000	0.083	0.195	Supported
BA →IA	0.297	0.296	0.032	9.253***	0.000	0.233	0.361	Supported
CBC →BA	0.567	0.570	0.029	19.783***	0.000	0.514	0.622	Supported
CR→ BA	0.299	0.301	0.031	9.516***	0.001	0.242	0.361	Supported
CR→ CBC	0.257	0.260	0.031	8.401***	0.000	0.193	0.314	Supported
CS→ CBC	0.267	0.268	0.034	7.748***	0.001	0.242	0.361	Supported
C*CSR→IA	0.039	0.041	0.042	0.932***	0.001	0.021	0.113	Supported
C*IA→PI	-0.020	-0.019	0.043	0.454***	0.652	-0.109	0.062	Not supported

*** p < 0.001.

## 5. Discussions and conclusions

One of the things that make people identify with a company is its attractive brand identity [6]. This phenomenon introduces a novel lens through which to examine relationship marketing, surpassing the conventional understanding that solely emphasizes the company’s vested interest in fortifying connections. This is due to the customer’s inclination towards enhancing their affiliations with the company.

This study examines the impact of business affiliations and consumer support for CSR initiatives on consumers’ perception of company image and reputation. The study contributes to the understanding of consumer-brand interactions and sheds light on potential factors that could enhance marketing partnerships. Consumers exhibit articulate and fervent communication regarding the brands and enterprises that have acquired a distinct position in their lives, as they possess a heightened emotional connection to certain entities compared to others [137]. Consumers resort to businesses and brands in order to satisfy their societal and personal requirements. Organizational attachment fosters a positive social identity by enhancing the congruence between an individual’s self-representation and the company’s self-definition [138]. This novel perspective is grounded on the Social Exchange Theory, a theoretical framework that posits that relationships can be comprehended through the lens of exchange [139]. Relationship satisfaction occurs when benefits exceed expenses as compared to expectations [140], and the consumer displays a strong inclination to willingly engage in mutually beneficial associations with the company. One of the primary benefits lies in the satisfaction of self-definitional needs.

The findings of our study indicate that CSR initiatives have influence on company IA, supporting earlier studies that discovered a connection between social activities and positive emotional, cognitive, and behavioral responses in consumers [13, 65, 66]. An essential element for the existence of intimate relationships is a positive emotional component, which holds particular significance in the association between CSR and IA [141, 142].

The identity consumption theory’s tenets are plainly demonstrated by the CBC mediation effect: when corporate and consumer identities are genuinely compatible, people should be able to define themselves more fully and explicitly by purchasing the corporate brand [143]. Consumers could feel more connected to companies that allocate a portion of their operations to promoting social well-being in the current climate, where the media constantly highlights its significance. Customers also find a company that participates in social events more appealing because of their desire to share with that company, which is a personality trait. These findings match earlier study, which found that a strong fit between organizational principles and members’ beliefs affected members’ inclination to stay in the organization [144, 145]. Given that customers desire a connection to the brand (IA) through product usage.

The effects of corporate affiliations on BA [65] and the model of [66], which laid the groundwork for the two-step mediation effect on BA by demonstrating the influence of CSR on BA through C-B congruity. We analyze the mediation effect through the indirect effects used in PLS-SEM bootstrapping. The concept of the indirect impact posits that in addition to the emotional appeal linked to a product, there are other types of value, such as social support, that may not be directly tied to a company’s primary production operations, but can nevertheless influence consumers’ impressions. This suggests that consumers are inclined to create well-informed and favorable evaluations of a brand’s image as a result of the company’s CSR endeavors. This indicates that CSR initiatives have the potential to impact consumers’ impressions of a brand in multiple dimensions, extending beyond the product itself.

The model definition backs up the concept that both parties the business and the customer contribute to their congruity, for example, by participating in CSR initiatives and having their efforts recognized by the public. Congruity between consumer and brands tends to need similar and significant involvement from both sides (the corporation through CSR actions and the consumer through CSR trust and support).

Our research adds to the discussion on the function of business partnerships in the consumer-brand relationship. In general, studies conducted over the past ten years have discovered that both kinds of relationships have an impact on BA [65]. Our research demonstrates that CSR has a substantially greater influence on business IA. This could be the effect of heightened competition brought on by the market’s declining selection of CR-based products. By enhancing consumer connections, which promote greater customer loyalty, favorable word-of-mouth, and other advantages, businesses use CSR initiatives to boost their capability to contest in the market. CR may have evolved into a benchmark, below which businesses struggle to survive in the market, and beyond which businesses get competitive advantages in the form of connections as a consequence of CSR efforts. An international public health event called COVID-19 reaffirms the favorable effect between CSR and identity attraction and interestingly the effect between identity attraction and purchase intention was negative.

### 5.1. Contributions and theoretical implications

Our study support the results of Zhang, 2022 [104], Who claimed that COVID-19 has no effect in the relationship between CSR and loyalty. The main rationale for this phenomenon is that amongst the COVID-19 pandemic, consumers had adverse effects and endured a prolonged period of fear and apprehension regarding their employment, professional endeavors, and prospects. This is the reason why they were unwilling to pay a higher price for corporations that engage in corporate social responsibility practices. However, consumers maintained the belief that CSR is a crucial obligation for firms during challenging periods, serving to safeguard individuals from pandemics, preserve the environment, and promote public health. By implementing this strategy, enterprises have the potential to enhance customer satisfaction and effectively draw more consumers to their establishment. Our research aligns with earlier work of [54], which showed how CSR affects identity attractiveness and company identification. The authors suggested that there is a positive correlation between these two factors, which consequently influences purchase intention.

Our research entails the development and empirical examination of a theoretical framework that explores the underlying factors contributing to IA. This framework draws upon established theories of social identity and organizational identification. The findings indicate that CSR exerts a more significant impact compared to Corporate Reputation (CR), potentially attributable to heightened competition and reduced heterogeneity based on CR within the market. Additionally, they provide empirical evidence supporting the association between IA and customer perceptions of business relationships.

The theoretical significance of this study lies in its examination of the factors that come before the attraction of firm identity attraction (IA) in consumer-business interactions, while considering the COVID-19 pandemic as a moderating factor. This study aims to improve understanding of the factors that contribute to identity attraction (IA) by developing a validated framework based on well-established theories of social identity and organizational identification. Furthermore, the aforementioned scholarly article provides a comprehensive analysis of the significant impact of CSR on identity attraction (IA), taking into account the moderating effect of the COVID-19 pandemic on this relationship. The findings of the research suggest that while the presence of COVID-19 may not directly influence individuals’ purchasing intentions, it does enhance the association between CSR and brand attraction. The present research makes a significant scholarly contribution by shedding light on the complex dynamics between organizational identity, CSR, the COVID-19 pandemic, and consumer behaviors.

### 5.2. Managerial implications

These conclusions have immediate ramifications for marketing professionals. When communicating with their stakeholders, businesses commonly present themselves as either a wonderful CR or CSR organization [14]. Strengthening the consumer-brand relationship requires highlighting the significance of the company’s non-product elements, such as its values and characteristics, its social responsibility initiatives, and the networking opportunities it provides [6]. In order to emphasize non-product elements and comprehensively evaluate consumer perceptions of CSR, businesses have the ability to employ a variety of strategies. Firstly, organizations have the opportunity to demonstrate their CSR initiatives through a range of communication channels, including social media platforms, website content, and press releases. Businesses have the ability to cultivate trust and captivate consumers by effectively communicating their dedication to social and environmental causes through the utilization of narratives and visual representations. Furthermore, the incorporation of CSR into the brand’s identity and values can yield significant impact. By prioritizing transparency, ethical sourcing, and sustainable practices in all aspects of business operations, a coherent and unwavering message is conveyed to consumers. Furthermore, engaging in collaborations with non-profit organizations or establishing partnerships for community projects can enhance the business’s social impact. Finally, the proactive pursuit of feedback from consumers via surveys, focus groups, or online forums can yield valuable insights and facilitate the customization of CSR initiatives to align with their expectations and preferences. Through the consistent emphasis and prioritization of non-product aspects, as well as the demonstration of sincere dedication to CSR, businesses have the ability to cultivate favorable consumer perceptions and establish enduring relationships founded on mutual values. In order to maximize the chance of developing long-lasting relationships with customers through identification, a business must represent and transmit its identity by offering both CSR and CR information, as well as measuring consumer support for CSR operations. As a result, companies may locate more receptive audiences and provide this information quickly and effectively.

It’s also important to consider how consumers see the reasons behind CSR initiatives [146], discovered that CSR attritional judgments affected purchase intent, raising issues with impression management and subjective bias [83]. If consumers hold pre-existing beliefs that businesses engage in CSR activities primarily for the purpose of enhancing their public image rather than out of genuine ethical considerations, then the effectiveness of communicating CSR initiatives as a marketing strategy may diminish. The resolution of this issue could potentially be achieved by considering wider societal expectations and requirements during the process of policy formulation and decision-making [147]. This broader perspective will help to lessen consumer mistrust since everyone will be aware of how businesses deal with CSR practices and how they interact with their social environment.

### 5.3. Limitation and future research

This study has made several important discoveries, but it also has certain limitations. First, we examined how individuals felt about a small number of corporations, so we should exercise caution when applying the results to situations in which customers purchase comparable goods (financial services) from a large number of businesses. Future experimental manipulations of business relationships, balancing corporate social responsibility support, CBC, and customer assessments of product quality among diverse firms may serve to substantiate the conclusions of this study. Second, despite the fact that we focused on IA motivated by corporate social responsibility actions, consumers also associate with companies based on other variables like the industry (for example, athletes and celebrities). The transfer of identification can be greatly impacted by a range of factors, such as the perceived similarity between domains and the degree of emotional investment. Consumers establish connections with companies through various factors, one of which pertains to the adoption and execution of corporate social responsibility initiatives. Nevertheless, the extent to which consumer identification, initially cultivated within a particular industry, such as athletics, can be applied to another industry, specifically CSR, remains uncertain at present. Understanding this transaction is of utmost importance, as it holds the potential to propose innovative collaborative marketing partnerships alongside corporate social responsibility endeavors. Further research in this particular field will yield valuable insights regarding the dynamics and potential for knowledge dissemination among diverse industries.

It’s noteworthy that corporate social responsibility has been evaluated utilizing the Brown and Dacin [65] scale, using a concept of corporate social responsibility that has previously been developed. This strategy has been followed by other marketing researchers [14, 148], it suggests that consumers’ assessments of CSR when examining the scale’s sections might not accurately reflect how they understand these obligations [149]. The next step is to conduct preliminary research to identify the categories of social responsibilities that customers reflect when rating a certain business. Research findings from that initial assessment will always show corporate social responsibility in accordance with how consumers see that industry or firm. Further study should also be conducted in the context of financial performance of firms in the hard time of pandemic as customers were not willing to pay premium because of their financial insecurity.

Further investigation is warranted to explore the intricate methods by which CSR impacts business Identity Attraction (IA). What are the mediating variables or processes that influence or determine the nature of this relationship? For instance, does the perception of CSR efforts have an impact on consumers’ impression of a company’s values and mission, therefore influencing their identity attraction (IA)? Investigating these mechanisms can yield a greater level of elucidation. The present investigation predominantly employed a questionnaire survey and structural equation modeling as its methodological framework. However, it is recommended that forthcoming studies adopt a multi-method approach, integrating both qualitative and quantitative methodologies, in order to enhance the comprehensiveness and depth of the research. Qualitative research can provide valuable insights into the intricate dimensions of customer perceptions and behaviors pertaining to CSR and identity attraction. In addition to the impact of COVID-19, there are other external elements that may potentially modify the association between CSR and IA. Subsequent investigations may delve into the examination of these prospective moderating elements, including economic circumstances, industry-specific events, or shifts in cultural dynamics.

The study’s findings may have limited generalizability as a result of its narrow focus on industrial cities in Pakistan. Consumer perceptions and behaviors may be influenced differently in this region due to its distinct cultural and contextual factors, in comparison to other regions or countries. Furthermore, given that the study is dependent on data obtained through a questionnaire survey, it is important to acknowledge the potential presence of biases or limitations that may impact the accuracy of the responses. Additional investigation utilizing varied samples and alternative research methodologies would be advantageous in augmenting the generalizability of the results. However, the research paper offers significant contributions in understanding the correlation between CSR, information asymmetry (IA), and the moderating influence of the Covid-19 pandemic within the framework of consumer-business interactions.
